# Live Attenuated Vaccine Based on Duck Enteritis Virus against Duck Hepatitis A Virus Types 1 and 3

**DOI:** 10.3389/fmicb.2016.01613

**Published:** 2016-10-10

**Authors:** Zhong Zou, Ji Ma, Kun Huang, Huanchun Chen, Ziduo Liu, Meilin Jin

**Affiliations:** ^1^State Key Laboratory of Agricultural Microbiology, Huazhong Agricultural UniversityWuhan, China; ^2^College of Veterinary Medicine, Huazhong Agricultural UniversityWuhan, China; ^3^Key Laboratory of Development of Veterinary Diagnostic Products, Ministry of AgricultureWuhan, China; ^4^College of Life Sciences, Huazhong Agricultural UniversityWuhan, China

**Keywords:** duck hepatitis A virus type 1, duck hepatitis A virus type 3, duck enteritis virus, VP1, 2A-element, vaccine

## Abstract

As causative agents of duck viral hepatitis, duck hepatitis A virus type 1 (DHAV-1) and type 3 (DHAV-3) causes significant economic losses in the duck industry. However, a licensed commercial vaccine that simultaneously controls both pathogens is currently unavailable. Here, we generated duck enteritis virus recombinants (rC-KCE-2VP1) containing both VP1 from DHAV-1 (VP1/DHAV-1) and VP1 from DHAV-3 (VP1/DHAV-3) between UL27 and UL26. A self-cleaving 2A-element of FMDV was inserted between the two different types of VP1, allowing production of both proteins from a single open reading frame. Immunofluorescence and Western blot analysis results demonstrated that both VP1 proteins were robustly expressed in rC-KCE-2VP1-infected chicken embryo fibroblasts. Ducks that received a single dose of rC-KCE-2VP1 showed potent humoral and cellular immune responses and were completely protected against challenges of both pathogenic DHAV-1 and DHAV-3 strains. The protection was rapid, achieved as early as 3 days after vaccination. Moreover, viral replication was fully blocked in vaccinated ducks as early as 1 week post-vaccination. These results demonstrated, for the first time, that recombinant rC-KCE-2VP1 is potential fast-acting vaccine against DHAV-1 and DHAV-3.

## Introduction

Duck virus hepatitis (DVH) is an acute, highly contagious, and rapidly fatal disease of young ducklings usually less than 4 weeks of age. This disease is characterized primarily by ecchymotic hemorrhage and liver necrosis. DVH is mainly caused by duck hepatitis A virus (DHAV). DHAV, which belongs to the family *Picornaviridae* and genus *Avihepatovirus*, was first described in the USA in 1949. DHAV strains are categorized into three different serotypes: the traditional serotype 1 (DHAV-1; Levine and Fabricant, 1950; Asplin, 1965; Kim et al., 2006; Ding and Zhang, 2007), a serotype only reported in Taiwan (DHAV-2; Tseng and Tsai, 2007), and a novel serotype isolated in China and South Korea (DHAV-3; Fu et al., 2008; Xu et al., 2012). No cross-neutralization reaction between DHAV-1 and DHAV-2 (Tseng et al., 2007) and limited cross-neutralization reaction between DHAV-1 and DHAV-3 (Kim et al., 2007) have been reported. In mainland China, DVH is primarily caused by DHAV-1 and DHAV-3, and infection by DHAV-2 has not been documented to date (Kim et al., 2007; Fu et al., 2008).

As a member of *Picornaviridae*, DHAV is a small, non-enveloped virus with a single-stranded, positive-sense RNA genome approximately 7800 nucleotides in length. The whole open reading frame (ORF) encodes three mature structural proteins, namely, capsid proteins 0 (VP0), 1 (VP1), and 3 (VP3). The ORF also encodes nine non-structural proteins (A1–2A2–2A3–2B–2C–3A–3B–3C–3D). Among these proteins, the major surface protein VP1 is the principal antigenic determinant that plays an essential role in pathogenicity, evolution, and virulence (Jin et al., 2008; Liu et al., 2008; Li C. et al., 2013; Zhang et al., 2015). Therefore, VP1 is a potential target for vaccine and drug development.

Co-infection with DHAV-1 and DHAV-3 has recently become increasingly frequent in domestic ducks, resulting in major economic losses to the duck industry in China and Korea (Chen et al., 2013; Chen L. et al., 2014; Soliman et al., 2015). However, no commercial DHAV vaccine is presently available to simultaneously control both DHAV-1 and DHAV-3. Obviously, alternative strategies for developing vaccines to prevent co-infections by DHAV-1 and DHAV-3 are urgently needed.

Duck viral enteritis, which is caused by infection with the virulent duck enteritis virus (DEV), is a highly serious infectious disease in duck (Li Y. et al., 2009). Vaccination combined with strict biosecurity practices has been the recommended approach for controlling DEV infection. Attenuated DEV of the C-KCE strain from embryonated chicken egg has been routinely used as live vaccine in ducks for over half a century without safety concerns for humans and any other animals (Liu et al., 2011; Wu et al., 2012). Additionally, C-KCE vector offers the advantage of efficiently generating both humoral and cellular immune responses (Shawky and Schat, 2002). More importantly, C-KCE is stable, efficacious, and cost effective to produce. Additionally, the vector overcomes pre-existing antibodies (Liu et al., 2011).

In recent years, the C-KCE vaccine strain has been developed as a vector for expressing foreign antigens for vaccine purposes. Previously, our group successfully generated C-KCE strain-based recombinant viruses delivering the hemagglutinin (HA) gene of avian influenza virus H5N1 as a bivalent vaccine for protecting of ducks against H5N1 and DEV challenges (Zou et al., 2015). The robust protection afforded by the C-KCE-H5HA vaccine against a lethal H5N1 challenge raises the possibility that the C-KCE vector will prove useful not only for avian influenza virus but also for other viruses in ducks. In the present study, to explore this hypothesis, we further engineered the C-KCE vector to express two different types of VP1 from DHAV-1 and DHAV-3. We also evaluated the ability of the recombinant C-KCE to protect ducks from DHAV-1 and DHAV-3 challenge.

## Materials and Methods

### Ethics Statements

All of the animal experiments were approved by the Research Ethics Committee, Huazhong Agricultural University, Hubei, China (HZAUMO2015-0015). All the animal experiments were carried out in accordance with the recommendations in the Guide for the Care and Use of Laboratory Animals from Research Ethics Committee, Huazhong Agricultural University, Hubei, China.

### Virus Strains and Cells

The attenuated DEV C-KCE vaccine strain, obtained from the China Institute of Veterinary Drugs Control, was propagated and titrated in chicken embryo fibroblasts cells (CEFs) cultured in M199 medium (Gibco) supplemented with 10% fetal bovine serum (FBS) and 1% antibiotics.

DHAV-1 (JX-1) (GenBank accession number: EF093502.1) and DHAV-3 (Hubei 1302) (GenBank accession number: KJ744260.1) were propagated in the allantoic cavities of 10-day-old specific-pathogen-free (SPF) embryonated duck egg. The allantoic fluids were collected and stored at -80°C.

### Generating the Recombinant Virus rC-KCE-2VP1

**FIGURE 1:**
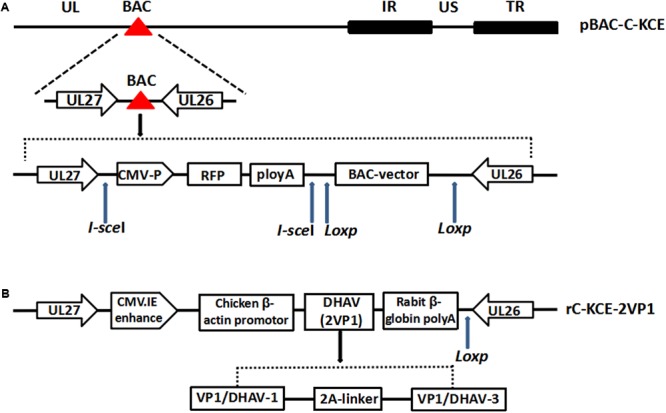
**Insertion of foreign genes into the C-KCE genome. (A)** Full-length C-KCE clone harboring mini-F plasmid with the red fluorescent protein as a selection marker. **(B)** Generation a duck enteritis virus (DEV) recombinants (rC-KCE-2VP1) containing two types of VP1/DHAV (duck hepatitis A virus)-1 and VP1/DHAV-3 between UL27 and UL26 with a self-cleaving 2A-element.

We previously established a system to regenerate C-KCE by a combined bacterial artificial chromosome (BAC) and mating-assisted genetically integrated cloning (MAGIC) strategy (**Figure 1A**). This strategy allowed us to achieve stable insertion of the HA gene from H5N1 between the UL26 and UL27 genes of C-KCE without altering the replication and immunogenicity of the parental virus (Zou et al., 2015).

To construct the rC-KCE-2VP1, the gene fragment (VP1/DHAV-1 + 2A + VP1/DHAV-3) was synthesized by Sangon Biotech Life Science Products and Services as outlined in Supplementary Figure 1. Subsequently, the fragment was cloned into the *BamH* I and *EcoR* I sites present in pRThGA1 to generate the donor vector plasmid pRThGA1-2VP1, whereas the self-cleaving 2A peptide of FMDV acted as a labile linker between the two genes VP1/DHAV-1 and VP1/DHAV-2. Details of the methods used for MAGIC-mediated recombineering are provided elsewhere (Zou et al., 2015). A rC-KCE-2VP1 construct without the BAC vector was also generated as described previously (Wang and Osterrieder, 2011).

### Confirmation of Expression of Two Different Types of VP1 in CEFs Infected with rC-KCE-2VP1

Expression of two different types of VP1 protein in rC-KCE-2VP1 was evaluated by immunofluorescence (IFA) and Western blot. For IFA, the CEFs grown on coverslips in six-well plates were infected at an MOI of 1 with rC-KCE-2VP1 or C-KCE. Monoclonal antibodies (mAb) against VP1/DHAV-1 and VP1/DHAV-3 (previously prepared in our laboratory) were used as primary antibodies. Details of the methods used for produce mAb against VP1/DHAV-1 and VP1/DHAV-3 are provided elsewhere (Yang et al., 2011). Briefly, adult female BALB/c mice were injected with purified VP1/DHAV-1 or VP1/DHAV-3 protein with adjuvant. The secondary antibodies were fluorescein isothiocyanate-labeled goat anti-rabbit IgGs (Santa Cruz Biotechnology, Santa Cruz, CA, USA). The CEFs nuclei were then stained with 4′-6-diamidino-2-phenylindole (DAPI). The cells were observed under a fluorescence microscope (Carl Zeiss, Germany). For Western blot analysis, VP1 expression was analyzed in CEFs in six-well plates infected with rC-KCE-2VP1 and C-KCE at an MOI of 1. mAb against VP1/DHAV-1 and VP1/DHAV-3, mAb (the same mAb against VP1/DHAV-1) against 2VP1, Polyclonal antibodies (pAb) against gB (previously prepared in our laboratory), and mAb against GAPDH (Santa Cruz Biotechnology, Santa Cruz, CA, USA) for the control were used as primary antibodies. Goat horseradish peroxidase (HRP)-conjugated anti-rabbit or anti-mouse IgGs were used as secondary antibodies. The bands were visualized using ECL detection reagents (Thermo, USA) in accordance with the manufacturer’s instructions.

### Animal Experiments

Specific-pathogen-free ducks were obtained from the Harbin Veterinary Research Institute, China. A total of 387 1-day-old SPF ducks were adopted for our studies. Three animal experiments were conducted to evaluate the immunogenicity and protective efficacy of the rC-KCE-2VP1 vaccine against DHAV-1 and DHAV-3 challenges.

#### Experiment 1

To test the serological responses against VP1/DHAV-1 and VP1/DHAV-3 in ducks immunized with rC-KCE-2VP1, we randomly divided the ducks into three groups (five per group), each group receiving one immunization subcutaneously with 10^5^ PFU (recommended dose for DEV vaccine in the field) of rC-KCE-2VP1, C-KCE, or PBS as negative control. Serum samples were then collected from all the groups to evaluate serological responses at 3 days, 1, 2, 3, 4, and 5 weeks post-vaccination (pv).

#### Experiment 2

To evaluate the level of clinical protection provided by rC-KCE-2VP1 against DHAV-1 and DHAV-3, 312 ducks were randomly divided into 24 groups (13 per group). A total of eight groups of ducks were inoculated subcutaneously with 10^5^ PFU of rC-KCE-2VP1, and 16 groups were inoculated with 10^5^ PFU of C-KCE or PBS as negative control. The ducks were then intramuscularly challenged with 100 LD_50_ of DHAV-1 or DHAV-3 at 3 days, 1, 2, or 4 weeks pv. Three ducks in each DHAV-1/DHAV-3 virus-challenged group were then humanely sacrificed on day 2 post-challenge (pc), and duck organs, including liver, lung, spleen, kidney, and brain, were collected to determine viral titers.

#### Experiment 3

To measure the T-cell responses in the spleens of vaccinated ducks, 12 groups of ducks (five per group) were subcutaneously inoculated with rC-KCE-2VP1 (10^5^ PFU), C-KCE (10^5^ PFU), or PBS (control). At 3 days, 1, 2, 4, and 5 weeks pv, the ducks were sacrificed humanely. Their spleens were harvested to screen the cellular immune responses.

### Interferon-Gamma (IFN-γ) ELISpot Assay

T-cell responses were determined using an IFN-γ ELISpot assay (Li Z. et al., 2013). Briefly, duck spleens were homogenized and washed with Hank’s Balanced Salt Solution. Gey solution was then added to remove the red blood cells. Splenocytes purified from ducks in Complete Tumor Medium were added into a 96-well plate (seeded at 2 × 10^5^ cells/well) pretreated with 70% ethanol and coated with anti-duck IFN-γ mAb. Cells were restimulated with synthetic peptides derived from VP1/DHAV-1 or VP1/DHAV-3 as a specific antigens, respectively. The cultures were incubated under 37°C and 5% CO2 for 48 h and conducted in accordance with the manufacturer’s protocol (TSZ, USA). Spots were counted using an AID ViruSpot Reader (Cell Technology, Inc.). Results are presented as the mean number of cytokine-secreting cells subtracted by the mean number of mock stimulation per 10^6^ splenocytes.

### Serological Analysis of Duck Serum

Duck sera were harvested at different time points to evaluate the antibody levels by indirect enzyme-linked immunosorbent assay (ELISA) and neutralization test. For indirect ELISA, ELISA plates (Corning Costar) were briefly coated with purified His-tagged VP1/DHAV-1 or VP1/DHAV-3 proteins and incubated overnight at 4°C. After blocking, the plates were incubated with duplicate twofold serial dilutions of test sera for 1 h at 37°C. HRP-conjugated goat anti-rabbit IgG was used at 1:2000 dilution to detect bound antibodies for 1 h at 37°C. The wells were then rinsed with PBST and incubated with TMB. Substrate development was stopped by adding 2 mM sulfuric acid. Optical density (OD) was measured at 450 nm using a TECAN microtiter plate reader. For the neutralization test, all serum samples were mixed and inactivated at 56°C for 30 min. The neutralization test was then performed using 9-day-old SPF duck embryonated eggs as described previously (Song et al., 2014).

### RT-PCR Assay for the Detection of Viral Loads

Virus titers in heart, spleen, liver, kidney, and brain were determined by using a one-step real-time TaqMan RT-PCR assay (Yang et al., 2008). The set of primers and probes used in this research have been previously validated (Lin et al., 2016). The primers and TaqMan probe for DHAV-1 were DHAV-1F, DHAV-1R, and Probe 1. The primers and TaqMan probe for DHAV-3 were DHAV-3F, DHAV-3R, and Probe 2. DHAV-1TF, DHAV-1TR, DHAV-3TF, and DHAV-3TR (Supplementary Table 1) were designed to amplify the fragments of two standard templates. The fragments were then cloned into pGEM-T Easy vector in accordance with the instructions of the manufacturer (Promega). One-step, real-time TaqMan RT-PCR assays were carried out on an Applied Biosystems 7500 Fast real-time PCR system (Life Technologies, Carlsbad, CA, USA).

### Statistical Analysis

All experiments were reproducible and performed in triplicate. Statistical analyses were conducted by a one-way ANOVA test to compare the data of the difference groups using GraphPad Prism version 5.0 (GraphPad Software, La Jolla, CA, USA). *p*-values of <0.05 were considered statistically significant.

## Results

### Generation and Characterization of Recombinant C-KCE Containing the VP1/DHAV-1 and VP1/DHAV-3

A recombinant C-KCE carrying the HA gene of the influenza A/duck/Hubei/xn/2007 (H5N1) virus was previously generated by a combined BAC and MAGIC strategy (Zou et al., 2015). By using the same strategy, here we generated a recombinant C-KCE virus carrying the VP1/DHAV-1 and VP1/DHAV-3 inserted into the UL27 and UL26 gene junction, under the control of chicken β-actin promoter and cytomegalovirus immediate enhancer. We designated the resulting recombinant C-KCE virus as rC-KCE-2VP1 (**Figure 1B**).

**FIGURE 2:**
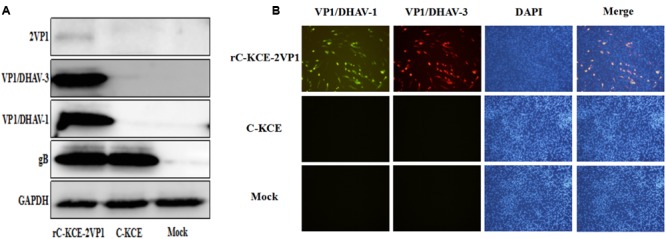
**Detection of VP1/DHAV-1 and VP1/DHAV-3 expression by rC-KCE-2VP1 in infected chicken embryo fibroblasts cells (CEFs). (A)** Detection of VP1/DHAV-1 and VP1/DHAV-3 protein expression in rC-KCE-2VP1-infected CEFs by Western blot. **(B)** Confirmation of the expression of VP1/DHAV-1 and VP1/DHAV-3 protein in rC-KCE-2VP1-infected CEFs by using immunofluorescence. CEFs infected with C-KCE or mock-infected CEFs were used as controls.

Expression of the inserted VP1 gene in the rC-KCE-2VP1-infected cells was determined by Western blot and IFA. Western blot analysis was performed on the whole-cell extract of rC-KCE-2VP1-infected CEFs. Protein bands of approximately 26 kDa (molecular mass of the VP1 protein of DHAV) were clearly visible in the rC-KCE-2VP1-infected cell extract, demonstrating that VP1/DHAV-1 and VP1/DHAV-3 were efficiently expressed (**Figure 2A**). Moreover, VP1/DHAV-1 protein was expressed at levels similar to that of VP1/DHAV-3. Meanwhile, the cleavage efficiency of the 2A element was incomplete (**Figure 2A**), which was consistent with a previous study in which the 2A element was found to mediate cleavage at about 90% efficiency (Li J. et al., 2009). As expected, no specific bands were observed in either mock (PBS)-infected or C-KCE-infected cell lysates.

The results of IFA matched well with those of Western blot analysis. Upon examining the rC-KCE-2VP1-infected CEFs stained with a mixture of anti-VP1/DHAV-1 and anti-VP1/DHAV-3 antibodies, both red and green fluorescence signals were observed by fluorescence microscopy. Notably, the red and green fluorescence signals co-localized to the same CEFs (**Figure 2B**). However, cells infected with PBS and C-KCE showed no red and green fluorescence (**Figure 2B**). Thus, these results confirm that both VP1/DHAV-1 and VP1/DHAV-3 proteins were robustly expressed with a C-KCE vector using the 2A-element.

### Induction of Antibody Response in rC-KCE-2VP1-Vaccinated Ducks

**FIGURE 3:**
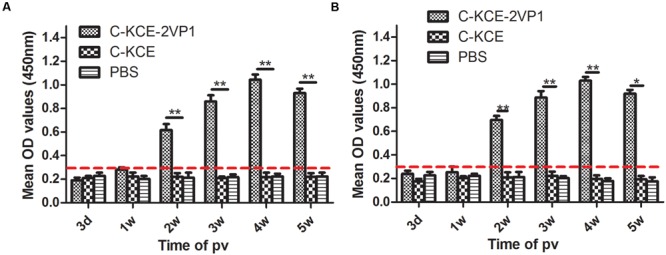
**Induction of anti-VP1 antibodies by rC-KCE-2VP1 in ducks.** Groups of five ducks were inoculated subcutaneously with 10^5^ PFU of rC-KCE-2VP1, C-KCE, or PBS (negative control). Sera were obtained at the indicated time points and pooled for detection of anti-VP1 antibodies by indirect ELISA. Serum samples were diluted in fetal bovine serum (FBS) at 1:50 for antibody detection. Each sample was independently tested twice. The result was obtained from mean ELISA absorbance values of three sera in each group. **(A)** Antibody responses were then assessed against the VP1/DHAV-1. **(B)** Antibody responses were assessed against the VP1/DHAV-3. The dashed line shows the detection limit for a positive response. Data are shown as the means ± SD. ^∗^ *P* < 0.05; ^∗∗^ *P* < 0.01.

To evaluate the immunogenicity of the recombinant rC-KCE-2VP1 vaccine, total anti-VP1/DHAV-1 and anti-VP1/DHAV-3 antibody responses were determined by an indirect ELISA. As shown in **Figure 3A**, antibody levels of the C-KCE and PBS-inoculated groups were lower and considered negative. In contrast to the antibody level in the control group, the earliest time point of detection of anti-VP1/DHAV-1 antibody in the rC-KCE-2VP1-vaccinated groups was week 2. In particular, the mean antibody level started to increase in week 3 but peaked with statistically significant difference compared to weeks 2 and 3 in week 4. However, the antibody level began to decline in week 5 (**Figure 3A**). The anti-VP1/DHAV-3 antibody responses were consistent with those observed for anti-VP1/DHAV-1 (**Figure 3B**).

**FIGURE 4:**
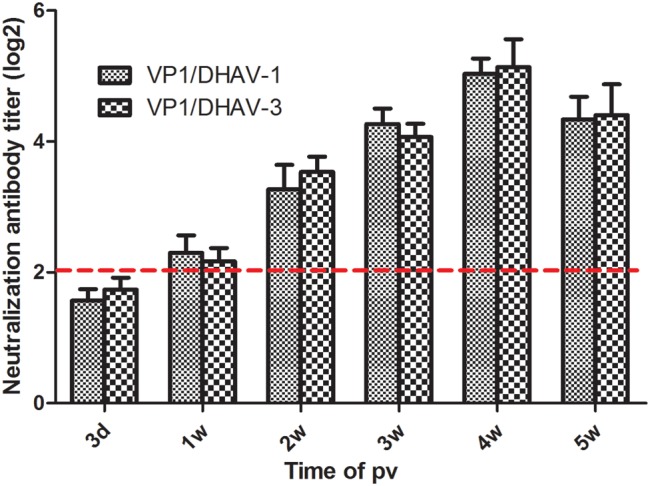
**Neutralization of antibody responses against both DHAV-1 and DHAV-3 induced by rC-KCE-2VP1 immunization in ducks.** Groups of five ducks were inoculated subcutaneously with 10^5^ PFU of rC-KCE-2VP1, C-KCE, or PBS (negative control). Blood samples were collected, and sera were prepared at the indicated time points to detect the NT antibody against DHAV-1 and DHAV-3 in nine-day-old specific-pathogen-free (SPF) duck embryonated eggs. NT antibody titers for ducks are expressed as log_2_. Dotted lines indicate the thresholds for a positive response.

Next, serum samples were further assessed using the neutralization test assay to detect the presence of neutralizing antibodies, a marker of immunogenicity, against both VP1/DHAV-1 and VP1/DHAV-3. No detectable neutralizing antibodies were observed against VP1/DHAV-1 and VP1/DHAV-3 tested in ducks that received mock vaccination during the entire experimental period (data not shown). On the other hand, all the ducks vaccinated with the rC-KCE-2VP1 vaccine seroconverted at 2 weeks pv, with mean anti-VP1/DHAV-1 neutralizing antibody titers of 2 log_2_. The neutralizing antibody titers reached up to 3 log_2_ at 2 weeks pv and reached a peak of 5 log_2_ at 4 weeks. However, the titers gradually declined since 5 weeks pv (**Figure 4**). In addition, the mean NA levels against DHAV-1 and DHAV-3 was not statistically significant. Overall, these results revealed that ducks vaccinated with rC-KCE-2VP1 could induce humoral immune responses simultaneously against DHAV-1 and DHAV-3.

### Cellular Response to rC-KCE-2VP1 Viral Vaccination

**FIGURE 5:**
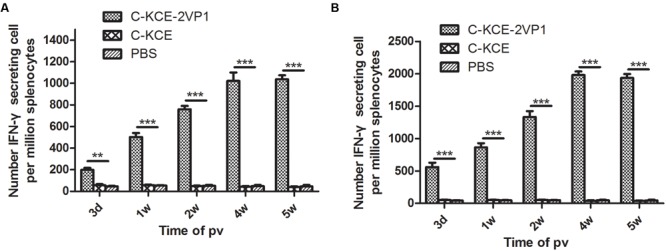
**rC-KCE-2VP1 viruses prime T-cell responses.** Groups of five ducks were inoculated subcutaneously with 10^5^ PFU of rC-KCE-2VP1, C-KCE, or PBS (negative control). VP1-specific responses of splenocytes obtained at 3 days, 1, 2, 4, and 5 weeks pv as determined by Interferon-Gamma (IFN-γ)-ELISPOT assay. **(A)** Splenocytes were restimulated with peptides derived from VP1/DHAV-1 as specific antigen. **(B)** Splenocytes were restimulated with peptides derived from VP1/DHAV-3 as specific antigen. Data are shown as the means ± SD. ^∗∗^ *P* < 0.01; ^∗∗∗^ *P* < 0.001.

Interferon-Gamma ELISpot assays were performed to evaluate whether rC-KCE-2VP1 can prime cellular immune responses. As expected, ducks that received the rC-KCE-2VP1 vaccine showed statistically significant increase in the number of IFN-γ-secreting cells in the spleen at each of the pv time points (3 days, 1, 2, 4, and 5 weeks), regardless of whether the spleen cells were stimulated with synthetic VP1/DHAV-1 or VP1/DHAV-3 peptide (**Figure 5**). Interestingly, the number of IFN-γ-secreting splenocytes stimulated with synthetic VP1/DHAV-3 peptide was significantly higher than those stimulated with VP1/DHAV-1 peptide. This was particularly notable at 4 weeks pv, when it reached an average peak intensity of one VP1/DHAV-3-specific T cell per 2,000 freshly isolated splenocytes (**Figure 5**). However, the efficiency of protection was not different based on subsequent challenge experiments. Thus, we speculated that the VP1/DHAV-3 peptide was more powerful antigen than the VP1/DHAV-1 peptide. By contrast, the mean number of IFN-γ-secreting cells in the C-KCE and PBS-inoculated groups that were extremely low (**Figure 5**) and considered negative was limited at any of the time points. These data indicate that rC-KCE-2VP1 immunization vigorously generated T-cell immune responses against both VP1/DHAV-1 and VP1/DHAV-3.

### Vaccine Efficacy against Pathogenic DHAV-1 and DHAV-3 Isolates Challenge in Ducks

Having shown that a strong T-cell immune response was rapidly induced after single-dose vaccination of rC-KCE-2VP1, we next questioned whether rC-KCE-2VP1 would confer protection against DHAV-1 and DHAV-3. Thus, the efficacy of rC-KCE-2VP1 as a candidate vaccine was evaluated by exposing of ducks to currently circulating isolates of pathogenic DHAV-1 and DHAV-3 after vaccination with rC-KCE-2VP1.

**FIGURE 6:**
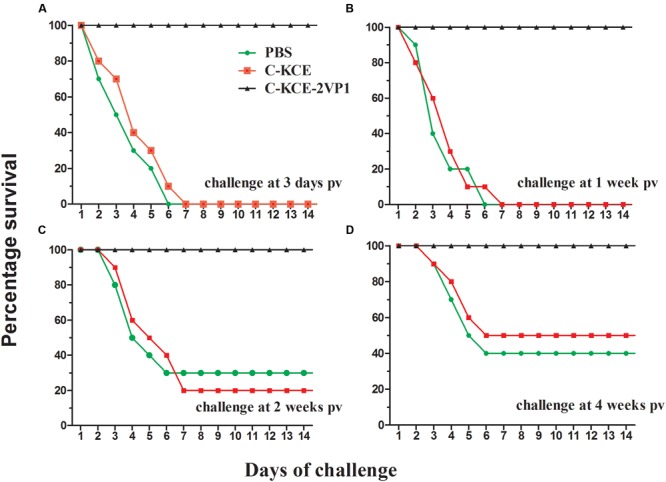
**Conferred protection from immunization of duck with rC-KCE-2VP1 vaccine against challenge with virulent DHAV-1 challenge.** Ducks were inoculated subcutaneously with 10^5^ PFU of rC-KCE-2VP1, 10^5^ PFU of C-KCE, or with PBS as control then intramuscularly challenged with 100-fold DLD_50_ DHAV-1 at 3 days **(A)**, 1 week **(B)**, 2 weeks **(C)**, or 4 weeks **(D)** pv, respectively. The ducks were monitored daily for 2 weeks after challenge.

**FIGURE 7:**
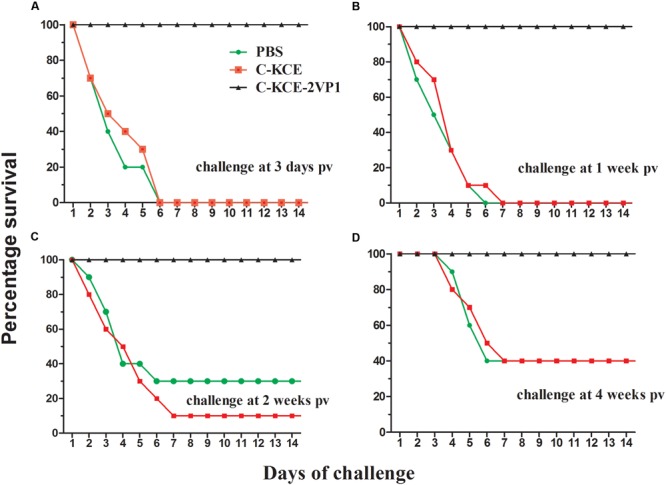
**Conferred protection from immunization of duck with rC-KCE-2VP1 vaccine against challenge with virulent DHAV-3 challenge.** Ducks were inoculated subcutaneously with 10^5^ PFU of rC-KCE-2VP1, 10^5^ PFU of C-KCE, or with PBS as control then intramuscularly challenged with 100-fold DLD_50_ DHAV-3 at 3 days **(A)**, 1 week **(B)**, 2 weeks **(C)**, or 4 weeks **(D)** pv, respectively. Ducks were monitored daily for 2 weeks after challenge.

Notably, all ducks in the rC-KCE-2VP1-vaccinated that had received DHAV-1 and DHAV-3 challenges survived at all time points pv (**Figures 6** and **7**). Furthermore, no clinical sign of disease was observed in the ducks given rC-KCE-2VP1 at any time point pv throughout the 14-day observation period, except for the ducks challenged on day 3 pv, which exhibited mild and transient symptoms at the start of the experimental period. These symptoms included slight loss of appetite and polydipsia. Conversely, in the ducks in the control groups C-KCE and PBS, mortality ranged from 50 to 100% (dependent on the days of ducks) with typical clinical features of appetite loss, wasting, ataxia, wryneck, and opisthotonus. The difference in protection efficacy between the two groups was not statistically significant. Thus, the rC-KCE-2VP1 candidate vaccine showed excellent protection against currently circulating isolates of pathogenic DHAV-1 and DHAV-3.

### Detection of Viral Load after DHAV-1 and DHAV-3 Challenge

To determine the effect of rC-KCE-2VP1 on viral replication, viral load in the liver, spleen, heart, kidney, and brain of ducks in each group were examined by the one-step real-time TaqMan RT-PCR assay. The challenge in the DHAV-1 virus was not recovered in any organs tested in the rC-KCE-2VP1 vaccinated ducks at weeks 1, 2, and 4 pv, and only low titers of challenge DHAV-1 virus were detected at day 3 pv, with average viral loads ranging from 10^2.68^ copies/g to 10^3.61^ copies/g (**Table 1**). On the contrary, in the PBS- and C-KCE-inoculated groups, DHAV-1 replication was detected in the livers at all four time points pv with high average viral loads ranging from 10^11.23^ copies/g to 10^9.49^ copies/g. Correspondingly, the mean viral loads ranged from 10^9.96^ copies/g to 10^8.94^ copies/g, 10^8.67^ copies/g to 10^7.58^ copies/g, 10^8.78^ copies/g to 10^6.98^ copies/g, and 10^8.15^ copies/g to 10^6.81^ copies/g in the spleens, hearts, kidneys, and brains, respectively (**Table 1**). The viral loads of DHAV-1 in the liver and spleen were significantly higher than those in other organs, which is consistent with previous research (Lin et al., 2016). Similar results were observed in the DHAV-3 challenge group (**Table 1**). Together, our data indicated that vaccination with rC-KCE-2VP1 completely blocked DHAV-1 and DHAV-3 replication in ducks as early as 1 week pv.

**Table 1:** Replication of challenge virus in ducks.

	Challenge time pv	Vaccine	Viral copy load in the organs in the ducks on 2 days pv (mean ± SD, log_10_ Copies/g)				
			Liver	Spleen	Heart	Kidney	Brain
DHAV-1	3 days	C-KCE-2VP1	3.28 ± 0.43	3.61 ± 0.26	2.46 ± 0.37	2.27 ± 0.46	2.68 ± 0.57
		C-KCE	10.26 ± 0.62	9.96 ± 0.32	7.58 ± 0.32	8.53 ± 0.43	7.56 ± 0.42
		PBS	9.87 ± 0.84	10.37 ± 0.41	8.59 ± 0.54	8.78 ± 0.31	8.13 ± 0.52
	1 week	C-KCE-2VP1	/	/	/	/	/
		C-KCE	11.23 ± 0.21	8.94 ± 0.76	8.59 ± 0.47	8.31 ± 0.39	7.94 ± 0.32
		PBS	9.64 ± 0.73	10.12 ± 0.76	8.67 ± 0.24	8.37 ± 0.52	7.86 ± 0.57
	2 weeks	C-KCE-2VP1	/	/	/	/	/
		C-KCE	9.91 ± 0.61	9.26 ± 0.49	8.31 ± 0.36	8.17 ± 0.39	8.15 ± 0.47
		PBS	10.41 ± 0.27	8.89 ± 0.73	7.64 ± 0.39	6.98 ± 0.56	6.81 ± 0.83
	4 weeks	C-KCE-2VP1	/	/	/	/	/
		C-KCE	9.49 ± 0.71	9.51 ± 0.42	7.98 ± 0.27	8.23 ± 0.31	8.11 ± 0.51
		PBS	10.17 ± 0.21	9.67 ± 0.52	8.43 ± 0.19	7.87 ± 0.43	7.67 ± 0.35
DHAV-3	3 days	C-KCE-2VP1	4.13 ± 0.26	3.72 ± 0.54	3.21 ± 0.33	2.47 ± 0.44	2.59 ± 0.53
		C-KCE	10.16 ± 0.63	10.43 ± 0.51	8.25 ± 0.63	8.59 ± 0.31	7.73 ± 0.63
		PBS	9.95 ± 0.66	10.26 ± 0.41	8.67 ± 0.31	7.74 ± 0.81	7.61 ± 0.57
	1 week	C-KCE-2VP1	/	/	/	/	/
		C-KCE	10.29 ± 0.43	10.33 ± 0.28	7.84 ± 0.61	8.37 ± 0.52	7.86 ± 0.51
		PBS	9.78 ± 0.62	9.54 ± 0.71	7.67 ± 0.72	7.82 ± 0.64	6.96 ± 0.74
	2 weeks	C-KCE-2VP1	/	/	/	/	/
		C-KCE	9.94 ± 0.72	10.21 ± 0.42	8.13 ± 0.35	7.59 ± 0.61	6.73 ± 0.53
		PBS	9.18 ± 0.52	9.71 ± 0.57	7.98 ± 0.47	8.13 ± 0.31	7.19 ± 0.62
	4 weeks	C-KCE-2VP1	/	/	/	/	/
		C-KCE	8.89 ± 0.74	9.53 ± 0.66	7.77 ± 0.61	8.43 ± 0.39	7.96 ± 0.43
		PBS	9.29 ± 0.51	9.91 ± 0.55	8.13 ± 0.43	7.97 ± 0.51	7.23 ± 0.33

*DHAV (duck hepatitis A virus)-1 and DHAV-3 replication in the organs of the ducks vaccinated with C-KCE-2VP1. Groups of three ducks were inoculated subcutaneously with 10^5^ PFU of rC-KCE-2VP1, C-KCE, or with PBS as control. Then, the ducks were challenged with DHAV-1 or DHAV-3 intramuscularly at 3 days, 1, 2, or 4 weeks pv. At day 2 after the challenge, the ducks were euthanized, and their organs were harvested for virus titration using a one-step, real-time TaqMan RT-PCR assay. Data are expressed as means ± standard deviations of log_10_. The backslash indicates that the challenge virus was not recovered at the corresponding time point.*

## Discussion

Duck virus hepatitis, mainly caused by DHAV, is a severe threat to the duck industry in Southeast Asia. Currently, China and Korea are severely affected by the epidemic duck hepatitis caused by DHAV-1 and DHAV-3. Moreover, mixed infections caused by DHAV-1 and DHAV-3 have become common in domestic ducks in eastern Asia. The increasing number of DHAV outbreaks in this region highlight the urgent need for effective control measures. Vaccination remains the most effective method that contributes to protecting ducks against DHAV-1 and DHAV-3 infection. Currently, modified live virus vaccines, which are attenuated by serial passages in chicken embryos, are available for controlling DHAV-1 infection in ducks (Woolcock and Crighton, 1979). However, no commercial vaccine is currently approved for use in ducks against the novel DHAV-3 strain. Developing a vaccine that simultaneously acts against both DHAV-1 and DHAV-3 is the most economical strategy for dealing with this crisis.

Attenuated DEV vaccine strains, including C-KCE and clone 03, have been used as vaccines over the past 50 years with proven track records. DEV vectors fulfill several important criteria of a promising vaccine vector in terms of efficacy, stability, and safety. To date, DEV as a vector vaccin has been extensively explored for use against H5N1 avian influenza virus and duck Tembusu virus (Liu et al., 2011; Liu X. et al., 2013; Chen P. et al., 2014; Zou et al., 2014). More importantly, DEV has also been used as a replicating vaccine in chickens to provide complete protection against the H5N1 influenza virus and the avian infectious bronchitis virus (Liu J. et al., 2013; Li et al., 2016), which greatly extends the application of DEV. However, DEV has not been evaluated as a vaccine vector for DHAV. Therefore, we sought to develop DEV as a vaccine vector for protection of ducks against DHAV infections. To this end, in this study, we generated a rC-KCE-2VP1 vector that delivered both VP1/DHAV-1 and VP1/DHAV-3. The two different types of VP1 separated by a 2A linker were inserted into the UL27 and UL26 gene junctions of C-KCE, which was proven not to alter the features of the parental virus C-KCE (Zou et al., 2015). We further evaluated the immunogenicity and protective efficacy against virulent DHAV-1 and DHAV-3 in SPF ducks. After single dose immunization, rC-KCE-2VP1 elicited humoral immune and cellular immune responses to VP1/DHAV-1 and VP1/DHAV-3. As early as 3 days pv, the ducks conferred solid protection against the DHAV-1 and DHAV-3 challenge.

In previous studies, different genes have been delivered either as separate ORFs (Fan et al., 1998; Szczypka et al., 1999) or bicistronic ORFs (Levenson et al., 1998; Klein et al., 1999; Martinez-Salas, 1999) incorporating the internal ribosome entry site (IRES). The first approach is time-consuming and laborious, whereas the second strategy can be limited by the dramatically reduced expression of the gene inserted downstream of the IRES (Roberts et al., 1998; Furler et al., 2001). Alternative strategies are needed to overcome these obstacles. The 2A element of FMDV, which encodes a mediator of primary polyprotein cleavage protease, has recently been used widely to link multiple genes in a single ORF under the control of a single promoter (Donnelly et al., 2001). Previously, the FMDV 2A sequence had been successfully incorporated in to retroviral (De Felipe and Izquierdo, 2000), lentiviral (Chinnasamy et al., 2006), and adeno-associated (Tan et al., 2010) vectors to construct multigene vectors. In this study, rC-KCE-2VP1 was developed to express the different type VP1 proteins by using 2A-element linkers between P1/DHAV-1 and VP1/DHAV-3. We observed that the two types of VP1 genes were efficiently expressed in rC-KCE-2VP1-infected CEFs. Moreover, the VP1/DHAV-1 protein was expressed at similar level as the VP1/DHAV-3 protein. Thus, recombinant rC-KCE-2VP1 was used to simultaneously express VP1/DHAV-1 and VP1/DHAV-3 by taking advantage of the FMDV 2A element. Utilizing 2A element, we also successfully constructed and obtained the C-KCE co-expression of the HA of H5N1, HA of H9N2, and E of duck Tembusu virus (data not shown).

Of note, single doses of 10^5^ PFU of rC-KCE-2VP1 induced rapid protection against both lethal virus challenges by DHAV-1 and DHAV-3 at 3 days pv. Virus replication was significantly reduced but not totally eliminated at 3 days pv, whereas virus replication was completely blocked at 1 week pv. This feature makes this vaccine extremely valuable, because duck hepatitis usually occurs as early as in less than 1-week-old ducklings. Generally, presence of antibody against the virus is a hallmark of protective immunity. Serological data revealed that both anti-VP1 specific antibodies and neutralizing antibodies were absent at 3 days pv. Similarly, a previous study has demonstrated that ducks that received attenuated vaccine DHAV-3 via intramuscular injection route conferred complete protection against DHAV-3 infection at 1 days pv (Kim et al., 2009). However, stimulating the antibody response at this time point pv may be exceedingly early. Hence, we can conclude that the DHAV antibody response does not necessarily correlate with the protection. Interestingly, the C-KCE vector itself triggers the production of interferons, which may play a role in early control of virus infection. However, interferon production triggered by C-KCE vector is unlikely to contribute to the rapid protection offered by the C-KCE-2VP1 vaccine because the control vector C-KCE did not offer any protection. Thus, T-cell responses, which are essential for virus clearance (Gao et al., 2006), are required for rapid protection. Accordingly, we analyzed the immunogenicity of rC-KCE-2VP1 and found that single dose vaccination induced vigorously T-cell immune responses to both VP1/DHAV-1 and VP1/DHAV-3 as early as 3 days pv. These results pointed that specific IFN-γ producing T cells might contribute to the early control of DHAV infection, as early as 3 days pv and at least until antibody responses appear, as previously shown for avian influenza infections (Zou et al., 2015). The mechanisms underlying for the rapid and complete protection afforded by the rC-KCE-2VP1 vaccine in such a short time requires further study. Further experiments are needed to address the question.

In Summary, we have demonstrated that rC-KCE-2VP1 can be used as a safe and effective candidate vaccine against DHAV-1 and DHAV-3 infections. rC-KCE-2VP1 elicited potent humoral and cellular immune responses, suggesting that C-KCE is a versatile vaccine platform for delivering the VP1 protein. This is the first study to show 100% protection against multiple DHAV strains using a single-vector single-injection vaccine. As such, rC-KCE-2VP1 is an excellent candidate trivalent live-attenuated vaccine.

## Author Contributions

Conceived and designed the experiments: ZZ and JM. Performed the experiments: ZZ, JM, and KH. Analyzed the data: ZZ, JM, HC, ZL, and MJ. Contributed reagents/materials/analysis tools: ZZ and MJ. Wrote the paper: ZZ, ZL, and MJ. All authors read and approved the final manuscript.

## Conflict of Interest Statement

The authors declare that the research was conducted in the absence of any commercial or financial relationships that could be construed as a potential conflict of interest.
