# Supplementary material for: Live Attenuated Vaccine Based on Duck Enteritis Virus against Duck Hepatitis A Virus Types 1 and 3
Source: Front Microbiol. 2016 Oct 10;7:1613. doi: 10.3389/fmicb.2016.01613 (PMC5056193; doi:10.3389/fmicb.2016.01613)
Supplement: Supplementary file 1 [file Presentation_1.PDF]

Supplementary Material

Live Attenuated Vaccine based on Duck Enteritis Virus against Duck Hepatitis A Virus Types 1 and 3

Zhong Zou*, Ji Ma*, Kun Huang, Huanchun Chen, Ziduo Liu
*** Correspondence:** Meilin Jin: jml8328@126.com

1 Supplementary Figures and Tables

1.1 Supplementary Figure 1

***GGATCC*(BamHI)GCCACCATG(kozak-sequence)**ggtgattctaaccagttgggggatgatgagccagtttgctttctaaattt
tgagacagctaatgtcccaatacaaggtgaatctcacactcttgttaagcacctgtttgggaggcaatggttagttaggactgtgcaacacgc
ttcaactgtgcaagagttggacctccaagttccagacagaggacatgcctctctcattcggttctttgcctatttttccggagagatcattcttac
cattgttaacaatggcactacaccagcaatggtagcacactcctattctatggatgacctcagttcagagtatgctgttacagcaatgggagg
tgtgatgattcctgctaacagtgccaaaaatatttctgtaccattctactctgtaacaccactcaggccaactcgaccaattcctggcacatcag
aggcaacttttggcagactgttcatgtggactcaatcaggaagtctttcagtttttatgggtctcaaaaagccagctctcttctttctactccctg
ctcccacctccacaatactatcacagaaatccaatgatgttattcccacattgaatcagtctggggatgaagtagattgtcacttctgtgaaatt
tgctctaaaatgaagaggaggaggaagccaagagggtacttcagattttgccttaggctcaaaacactagcatttgaactcaatctggaaat
tgaa**CAGCTGTTGAATTTTGACCTTCTTAAGCTTGCGGGAGACGTCGAGTCCAACCCTGGGCCC(2A-linker
from-FMDV)**ggtgattccaatcagcttggcgatgatgaaccagtgtgttttctcaattttgagactgcaaatgtaccaatacaaggggagt
cacacactttggtaaaacatctttttggtcgtcaatggctggttcgtactgttcaacatactagtgaggtacaagagttggatttgccagtacct
gatcagggtcacgcatctctgttgcgcttctttgcctacttctctggagaagtgattctgaccattgccaataatggaacaacaccatgcatggt
tgcacactcttatacaatggacaatctcacttctgaatatgctgtcactgccatggggggtattcttatcccagcaaactctgccaagaatatta
atattccattttattctgttacacctttacgccccacacgacccatgccagcatttcaggggggtggtttgacttttggcaggttgtatatttggac
acaatcaggaagcgtttctgtttttatgggcctccacaagccagctttgtttttccactacctgcaccaacttacacaacacacacacagttga
ataacattgaaaccatgaatctgcataatcaatcagatcagccagactgccccctgtgtaagatttgcaagaaaatgaagaaatggtctcgc
aaccatcgcccatttcgcttctgtttgagacttaaaacacttgcctttgagctccatctagaaattgaatag***GAATCC*(EcoR I)**

Supplementary Figure 1. The complete sequence of the gene fragment (VP1/DHAV-1 + 2A + VP1/DHAV-2) was synthesized by Sangon Biotech Life Science Products and Services. The names of the sequences are in bold and brackets after the sequence.

1.2 **Supplementary Table 1.** Primers used for quantifying the copy number of DHAV-1 and DHAV-3.

primer	Sequence (5'→3')
DHAV-1F	agacacatgttgctgaaaaact
DHAV-1R	tcccccttatacttaatgccag
Probe 1	FAM-atgccatgacactatctcatatgagtcagc-TAMR
DHAV-1TF	agacacatgttgctgaaaaact
DHAV-1TR	ccatcaccattctataagccac
DHAV-3F	cttgaacgtaatagagcttgg
DHAV-3R	agtcttttggtagagtcttagc
Probe 2	FAM-acacttggcaccattgtgtccctcataac-TAMRA
DHAV-3TF	acttgtagatgagatc
DHAV-3TR	gtccaattgagtaaacttaga
